# Sustainable Cellulose- and Pectin-Rich Triboelectric Nanogenerator for Mechanical Energy Harvesting and Self-Powered Humidity Sensing

**DOI:** 10.3390/polym17233130

**Published:** 2025-11-25

**Authors:** Seongwan Kim, Farhan Akhtar, Shahzad Iqbal, Muhammad Muqeet Rehman, Woo Young Kim

**Affiliations:** 1 Department of Electronic Engineering, Faculty of Applied Energy System, Jeju National University, Jeju 63243, Republic of Korea; pea8543@jejunu.ac.kr (S.K.); farhanakhtar@stu.jejunu.ac.kr (F.A.); shahzadiqbal@stu.jejunu.ac.kr (S.I.); 2 Faculty of Electrical Engineering, Ghulam Ishaq Khan Institute of Engineering Sciences and Technology, Topi 23640, Pakistan

**Keywords:** natural polymers, *Citrullus lanatus* rind powder (CLP), triboelectric nanogenerator (TENG), porous polymer films, self-powered humidity sensor

## Abstract

This study develops a high-performance triboelectric nanogenerator (TENG) through *Citrullus lanatus* rind powder (CLP) which originates from watermelon waste to generate sustainable power and detect humidity. The SEM and FTIR results showed that CLP contains a natural porous structure and multiple polar functional groups which improve both the charge transfer and retention capabilities. The CLP-TENG device operated in vertical contact–separation mode with PTFE as the counter layer to generate a 255 V open-circuit voltage and 30 µA short-circuit current and 35 µW peak power output at 4 Hz and 20 MΩ load. The device successfully charged a 4.7 µF capacitor to 5 V during a 80 s period and operated low-power electronic devices to prove its ability as a sustainable power source. The device output increased with increasing operating frequency while showing operation stability throughout more than 1000 cycles and seven days of continuous operation. The device demonstrated a strong humidity detection ability through its voltage response which decreased from 250 V to 120 V when the relative humidity rose from 30% to 90%. The research proves that agricultural waste can be transformed into environmentally friendly materials which perform well in green energy systems and environmental monitoring applications.

## 1. Introduction

The increasing global demand for sustainable energy solutions emerges from industrial growth and depleting fossil fuel reserves [1,2]. Meanwhile, the improper handling of agricultural waste products leads to environmental damage and greenhouse gas emissions [3]. The conversion of biowastes into energy-harvesting device materials could solve both problems by producing clean energy while reducing environmental contamination [4]. Organic waste contained substantial amounts of fruit peels and rinds with biodegradable polymers including cellulose, hemicellulose, lignin, and pectin, but these resources have not been fully explored [5,6,7,8,9]. The conversion of abundant biowastes into functional materials help to solve pollution problems while creating sustainable energy systems for circular economy operations [10].

The increasing need for sustainable energy solutions has driven the advancement of triboelectric nanogenerators (TENGs) [11,12,13,14,15,16,17,18,19]. The first TENG was reported in 2012, and researchers have since proven its operation through five different modes which generated electricity through mechanical energy conversion via triboelectricity and electrostatic induction. These five different approaches included the contact–separation mode (reported in this work also) [20], sliding mode [21], freestanding mode [22], single-electrode TENG [23], and dual-electrode TENG [24]. These energy-harvesting systems could generate small mechanical movements like walking/running, musical string vibrations, ocean waves, and wind currents [25]. The operation of TENGs depend on triboelectrification and electrostatic induction to generate electrical output from mechanical stimuli that can be achieved through using different material combinations. The lightweight design, affordable cost, and flexible nature of TENGs make them suitable for self-powered sensor applications, wearable electronics, and environmental monitoring systems [26]. The performance of TENGs could be enhanced by working on the three key factors mainly, including the surface charge density, effective contact area, and dielectric properties of triboelectric materials. In recent years, particularly over the past two years, researchers focused on utilizing natural and bio-derived materials with tailored surface characteristics and structures to develop environmentally friendly, high-performance TENGs. A few examples of such recent research works are discussed below.

Sarkar et al. [27] established a self-powered supercapacitor (SPSC) system which used biowaste-derived activated carbon for energy storage inside a TENG framework. Their developed system used two TENG units together with a carbon-based supercapacitor made from natural biowaste (abundant silk) to achieve direct energy harvesting and storage without needing external circuitry while showing the environmental benefits for sustainable power production and waste management. Chang et al. [16] established a biodegradable TENG using onion skins as a cost-effective and environmentally friendly triboelectric materials solution. The device solved the solid–solid biowaste TENG restrictions through its liquid–solid interface structure with a conductive electrode coating. The Rubiniu onion skin produced the highest output voltage of 800 V and power density of 0.8 mW/cm^2^ which enabled the operation of 375 LEDs. The ROS-TENG system operated for 10,000 cycles at 4 Hz and 50 N while showing outstanding durability to generate sustainable energy from agricultural waste. Prasanwong et al. [28] fabricated a high-performance TENG through the integration of activated carbon nanostructures from human hair biowaste (AC-HH) into a PVDF polymer. The PVDF@AC-HH TENG showed a better surface charge density and photoinduced charge generation because its graphene-like nanostructures in AC-HH enhanced both the charge transfer and storage capabilities. The device functioned as an efficient power source for small electronic components and a motion detection system capable of tracking finger movements. This study demonstrated an environmentally friendly approach to converting discarded human hair into photoactive materials for energy-harvesting applications.

Recognizing the significance of this research area, our group at the Smart Sensor and Device (SSD) Lab, Jeju National University (JNU), contributed to developing eco-friendly TENGs and self-powered sensors using natural biowaste materials to demonstrate their potential for sustainable energy harvesting and environmental monitoring. In successive studies, we explored various agro-waste materials to create active triboelectric layers and sensing components, including almond seed skin (EASS) [29], peanut skin powder (PSP) [30], and cellulose-enriched edible rice paper (CERP) [31], because these materials possessed biocompatibility, porous structures, and numerous surface functional groups which boost the charge transfer and adsorption capabilities. The EASS-TENG system produced a power output of 36.1 μW during 500 consecutive operation cycles. The EASS-based self-powered humidity sensor (SPHS) operated at a fast response/recovery time of 21/14 s while using its EASS material to power LEDs and wireless modules. The PSP-TENG system built from peanut skin waste produced 162 V of open-circuit voltage and 0.2 μA of short-circuit current, resulting in 2.2 mW of instantaneous power. The PSP-SPHS system operated with a 0.8 V/%RH sensitivity and responded within 4 s while recovering in 10 s. The CERP-based TENG system which used edible rice paper biowaste produced 162 V of output voltage while it produced 3 μW/cm^2^ of power density to successfully charge multiple capacitors and power 30 LEDs for three months.

In this research, *Citrullus lanatus* rind powder (CLP), derived from watermelon biowaste, was verified as a highly efficient and naturally abundant sustainable material for fabricating a triboelectric nanogenerator (TENG). The developed CLP-TENG served a dual purpose of efficiently harvesting mechanical energy into electrical energy and sensing environmental humidity without requiring any chemical treatment or surface modification. The developed device demonstrated an excellent electrical output performance, stable operation, and high sensitivity to humidity variations, highlighting its versatility as a self-powered system. Charging/discharging commercial capacitors and powering LEDs were also successfully demonstrated through CLP-TENG. The intrinsic porosity and rich polar functional groups (–OH and –COOH) of CLP enhanced the charge generation and transfer, enabling reliable energy conversion and environmental monitoring. This study introduced a cost-effective and eco-friendly approach for transforming agricultural biowaste into high-performance, multifunctional TENG devices, advancing the development of biodegradable and self-sustaining electronic systems within a circular energy framework.

## 2. Materials and Methods

**Figure 1:**
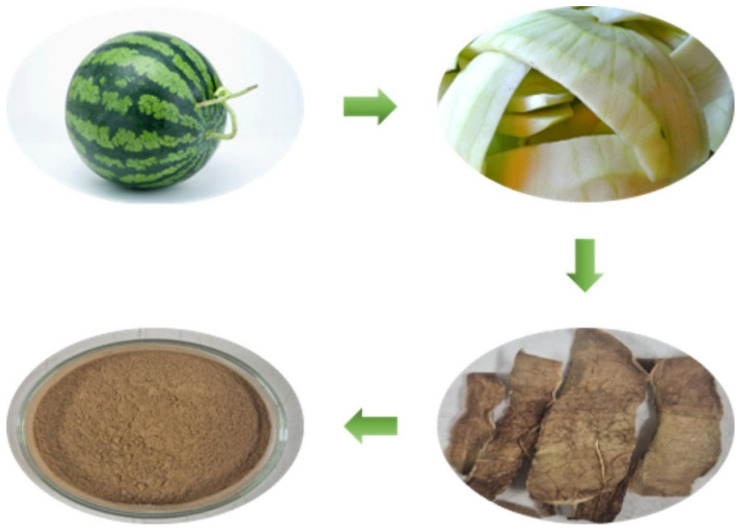
Processing steps of *Citrullus lanatus* rind powder (CLP) before using it as the tribo-positive layer of TENG.

Watermelon (*Citrullus lanatus*) was purchased from a local market in Jeju, Korea. The white inner rind of the watermelon was used, which was ground into watermelon rind powder (later used as tribo-positive layer). Copper (Cu) tape was used as the electrode material for fabricating the TENG device while polytetrafluoroethylene (PTFE) film was employed as the negative triboelectric layer. Polyethylene terephthalate (PET) was used as the substrate to support both the positive and negative layers of the TENG. All materials, including Cu tape, PTFE, and PET (purchased from 4Science, Seoul, Republic of Korea), were used without any further treatment. The optical images of obtained material after each processing step till obtaining CLP in powder form is shown in Figure 1.

**Figure 2:**
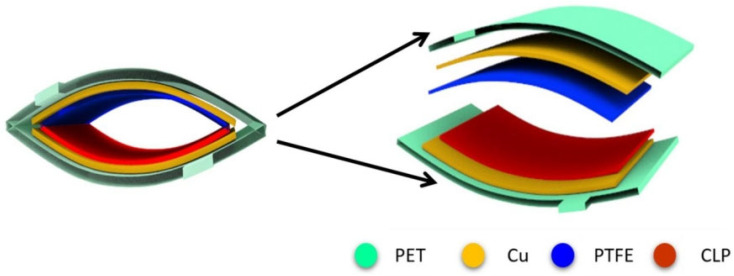
Layer-by-layer schematic diagram of CLP-TENG with color-coded labelling of each layer.

The inner juicy red part/flesh (mesocarp) and outer green skin (exocarp) of watermelon were removed to obtain only the white rind part. The obtained white rind was air-dried at room temperature (25 °C) before drying it at a higher temperature of 150 °C for 2 h. The dried rind was then processed further through grinding operations to produce fine *Citrullus lanatus* powder (CLP). The CLP powder served as the positive triboelectric material in the developed TENG device. PTFE was used as the negative triboelectric material due to its high electron affinity value (~3.3–3.8 eV). The combination of CLP and PTFE proved to be a highly efficient tribo-pair with Cu tape acting as both top and bottom electrode material for the developed TENG device. In short, conductive copper tape electrodes were mounted on PET substrates using double-sided adhesive. The exposed adhesive surface of each copper tape served as the attachment point for the tribo-active layers. PTFE film was affixed to the negative electrode, while CLP powder was uniformly spread and adhered to the positive electrode. The CLP layer in our device was not fabricated as a bulk film; rather, it consisted of a single particulate layer adhered directly to the adhesive surface of the copper electrode. Since no binder or slurry was employed, the powder naturally formed a monolayer coverage, as only one layer of particles could adhere to the adhesive. This configuration ensured robust contact and effective charge transfer between the triboelectric layers. The CLP-TENG device was designed to operate in the vertical-contact separation mode with an active surface area of 4 × 4 cm^2^ only. The complete schematic diagram of the developed TENG device with labeling of each formed layer is shown in Figure 2. The optical image of the developed CLP-TENG with proper labelling and the gap between its layers is provided in Figure S1. The reported outputs (in Section 3) represented the average of three independently fabricated devices, with variations within ±5%, demonstrating reproducibility. All chemical treatments, toxic solvents, and complex fabrication steps were avoided to preserve CLP’s natural porous structure, ensure environmental safety, and allow easy scalability. The minimal powdering process preserved the material’s open-cell structure, as confirmed by SEM, and enhanced its ion and charge attraction under humidity exposure.

The CLP films underwent SEM (Phenom Pharos G2 from Thermo Fisher Scientific in Seoul, Republic of Korea) analysis to study their surface structure and pore distribution. The FTIR (Alpha II from Bruker Optiks in Ettlingen, Germany) instrument scanned samples from 4000 to 400 cm^−1^ to detect their chemical functional groups. The CLP-TENG device underwent mechanical energy-harvesting tests through a precision-controlled linear motor which optimized its striking force, frequency, and distance settings. The digital oscilloscope (Keysight DSOX3014T from Keysight Technologies, Santa Rosa, CA, USA) and source measurement unit (Keysight 2911A from Keysight Technologies, Santa Rosa, CA, USA) recorded the output voltage and current signals. The impact force applied to the device was measured using a calibrated force testing instrument (Model JA-50 N, YILIDA, Linyi, China). The CLP-RHS humidity-sensing properties underwent evaluation through tests conducted in a highly controlled and customized humidity environment setup. In addition, energy-dispersive X-ray spectroscopy (EDS) revealed the presence of inorganic elements such as C, N, O, Mg, P, and K, which are the major constituents of the watermelon rind. The optical photograph of our customized experimental setup is shown in Figure S3 with each component labeled properly, followed by the optical image of the whole setup arrangement for powering LEDs in Figure S4.

## 3. Results

### 3.1. Morphological and Chemical Characterization

**Figure 3:**
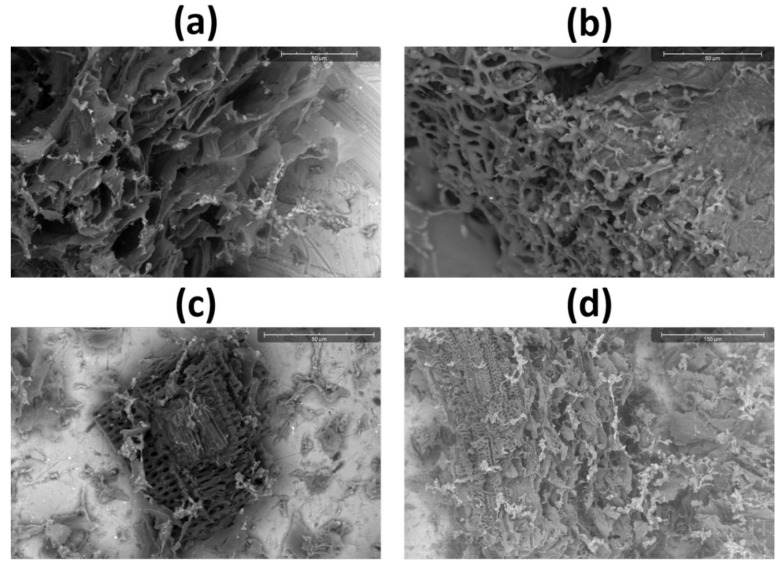
(**a**–**d**) SEM images of CLP taken at different resolutions (50 µm to 150 µm), clearly showing the fibrous and highly porous structure that is highly suitable for the high performance of both TENG and humidity sensing.

The SEM analysis showed that the dried watermelon rind powder maintained its natural porous structure which proved that the original internal structure of the natural material did not change after processing [32]. Such a structure with microvoids and fibrillar regions enhanced the surface area and facilitated moisture adsorption [33,34,35]. The SEM images show that CLP has a porous structure which provides both a high surface area and low weight for flexible deformation and efficient impact absorption through its internal air pockets as shown in Figure 3a–d. The CLP film displayed a cellulose fiber network that formed a sponge-like structure through its microvoids which was measured between. The device operates better because of its interconnected pores which create a greater contact surface area for better charge production and a higher triboelectric output. The flexible and lightweight nature of CLP enabled simple substrate attachment for building large-scale flexible biodegradable energy-harvesting devices. More similar SEM images of CLP at even higher resolutions (20 µm and 30 µm) are presented in Figure S2.

**Figure 4:**
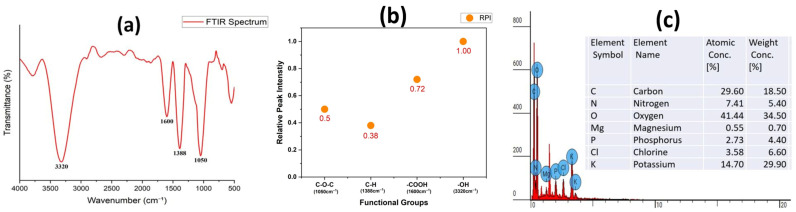
Chemical composition of CLP: (**a**) FTIR showing all characteristic peaks of O–H stretching bonds, C=O stretching bond, C–H bending, and C–O–C stretching bands; (**b**) normalized ATR-FTIR spectra of CLP showing relative peak intensity (RPI) analysis of key functional groups; and (**c**) EDS analysis of CLP showing its elemental composition in terms of wt% with C and O being the major contributors.

The FTIR spectroscopy technique helped to detect the specific functional groups which produce charges for triboelectrification and react with humidity. The wide absorption peak at 3320 cm^−1^ shows O–H stretching bonds from cellulose, hemicellulose, lignin, and pectin compounds [34,36]. The C=O stretching bond from lignin aromatic and phenolic groups produces a peak at 1600 cm^−1^ while the C–H bending and C–O–C stretching bands appear at 1388 cm^−1^ and 1050 cm^−1^, respectively. The FTIR spectrum verified that CLP consisted of a polysaccharide-based biomass containing hydroxyl and carboxyl groups that enabled triboelectric charge production and maintenance as shown in Figure 4a. These FTIR results align well with what was already reported by other FTIR studies on CLP [37,38,39]. The hydrophilic groups present in the CLP enabled dual operation as a triboelectric and humidity-sensing element. The surface functional groups stayed intact because no chemical treatment was used which guaranteed environmental safety and maintained stable operation and natural tribo-positive polarity. The combination of the fibrous–porous structure and high polysaccharide content made CLP an environmentally friendly dual-functional material for green electronic applications. The abundant –OH and –COOH functional groups in CLP readily adsorb water molecules, forming a thin hydration layer on the material surface. The abundant –OH and –COOH functional groups in CLP significantly increase the surface dipole density and provide electron-trapping sites, enhancing the charge retention under dry conditions. Under elevated humidity, these groups strongly interact with water molecules, forming a thin hydration layer that facilitates protonic and ionic conduction. This absorbed moisture enhances the mobility of naturally occurring ions (K^+^, Ca^2+^, and Mg^2+^), enabling humidity-dependent ionic conduction at elevated RH levels [40]. The increased ionic mobility raises the effective surface conductivity, which partially dissipates triboelectric charges and contributes to the observed reduction in output under high humidity. A secondary effect arises from dielectric modulation, as the hydration layer alters the local permittivity and partially screens surface charges, further influencing charge-transfer dynamics. Figure 4b shows the normalized ATR-FTIR spectra of *Citrullus lanatus* rind powder, showing the relative peak intensity (RPI) analysis of key functional groups. The intensities of O–H (≈3320 cm^−1^), C=O/–COOH (≈1600 cm^−1^), and polysaccharide-associated bands were normalized to the C–O–C stretching band at 1050 cm^−1^. The dominance of hydroxyl and carboxyl groups indicated a strong tribo-positive polarity and enhanced humidity responsiveness. This dual behavior of stabilizing triboelectric charges in dry air while promoting charge dissipation at high RH has already been widely reported for polysaccharide-rich biomass materials and underpins the humidity-responsive nature of CLP-based TENGs.

The EDS analysis further confirmed the elemental composition of CLP through its complementary data. The surface composition of CLP consisted mainly of O (34.5 wt%), K (29.9 wt), and C (18.5 wt%) with relatively smaller amounts of N (5.40 wt%), Cl (6.6 wt%), and P (4.4 wt%), and trace amounts of Mg (0.70 wt%) as shown in Figure 4c. The organic composition of cellulose, hemicellulose, and pectin in CLP was confirmed by the high carbon and oxygen content verified through EDS analysis which provides the necessary polar groups for surface charge development. The protein residues in the CLP contained nitrogen which creates additional sites for charge accumulation. The ionic conductivity of the CLP increased further when it absorbs water because of the presence of mineral salts including K^+^, Cl^−^, and PO_4_^3−^. The small amount of magnesium present in the material could affect the local dielectric properties which help maintain the charge stability between interfaces. The inherent ionic composition of CLP played a critical role in the humidity-modulated charge transport. Under elevated RH, the adsorbed water molecules form a continuous hydration layer within the porous structure, significantly enhancing the mobility of naturally occurring ions such as K^+^, Ca^2+^, and Mg^2+^. This increased ionic mobility promoted localized ionic polarization and elevated surface conductivity, which accelerated the charge dissipation and modulated the triboelectric output. Such behavior aligns with prior studies demonstrating that alkali and alkaline-earth metal ions in plant-derived polysaccharides markedly improve ionic conduction and humidity sensitivity. Therefore, the synergistic effect of the ionic species and hydrophilic functional groups in CLP underpins its strong humidity-responsive electrical characteristics. All elements present in CLP existed naturally and were harmless to the environment, which proves its safety as a green triboelectric material. The combined SEM, FTIR, and EDS analyses confirm that CLP possesses a porous polysaccharide–mineral structure with abundant polar and ionic sites. The combined properties of CLP provide ideal conditions for charge generation and humidity sensing, making it suitable for self-powered triboelectric and environmental sensors.

### 3.2. Electrical Output Performance of CLP-TENG

**Figure 5:**
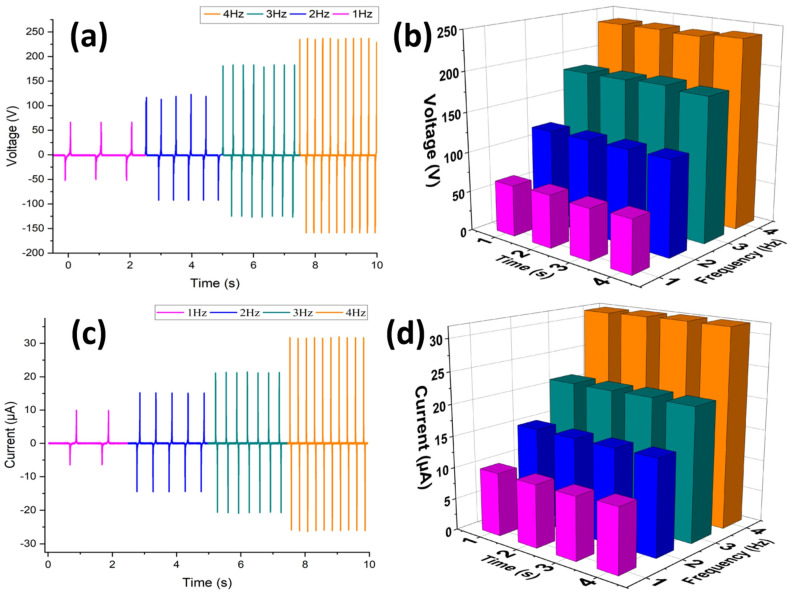
(**a**–**d**) Output voltage and current of CLP-TENG at different operating frequencies ranging from 1 Hz to 4 Hz with the electrical output increasing with increasing frequency.

A customized and programmable linear motor was used to evaluate the electrical performance of the CLP-TENG device by testing it in contact–separation mode at different frequencies, from 1 Hz to 4 Hz. The digital oscilloscope Keysight DSOX3014T and precision source meter Keysight B2911A were used to measure the open-circuit voltage (Voc) and short-circuit current (Isc). Increasing the number of contact–separation events per unit time resulted in higher voltage and current amplitudes, which resulted in better charge transfer and electrostatic induction. The results showed that the current levels increased with increasing frequency, i.e., from 10 μA at 1 Hz to 30 μA at 4 Hz, while the voltage levels increased from 60 V at 1 Hz to more than 250 V at 4 Hz. The frequency-dependent response demonstrated that the CLP matrix enabled efficient triboelectric charge generation and fast charge transport. The impact force applied to the device was measured as 28 N (at 4 Hz) using a calibrated force testing instrument (Model JA-50 N, YILIDA). Although the acceleration data could not be obtained, the measured force and controlled displacement ensure that the applied mechanical loading was consistent across the driving frequencies investigated. The comparative values of the output voltage and output current produced by CLP-TENG at different operating frequencies were shown in Figure 5a,b and Figure 5c,d, respectively. The individual responses of the output current and voltage of the developed CLP-TENG at different frequencies (for 10 s each) are presented in Figure S6 and Figure S7, respectively. We also tested the reproducibility of our device in this study by fabricating three similar CLP-based TENG devices under identical conditions and recorded their output performance whose graphical representation is provided in Figure S8. The revised manuscript now includes comparative graphs showing the open-circuit voltage and short-circuit current for all three devices. Additionally, the mean values with standard deviations (mean ± SD) are reported for both parameters, demonstrating consistent performance and confirming device reproducibility.

**Figure 6:**
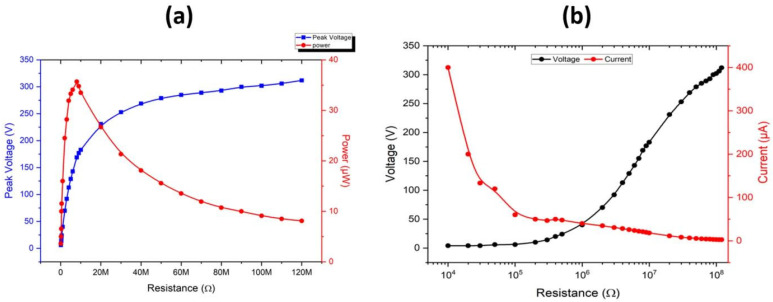
Electrical relationship of CLP-TENG: (**a**) maximum power analysis of CLP-TENG; and (**b**) non-linear behavior of current and voltage with different values of applied resistance that is a characteristic behavior of TENG devices.

This study also investigated how the electrical output changes with changing external load resistance, ranging from 0.1 MΩ to 120 MΩ. The output voltage increased while the output current decreased when resistance values increased in a pattern that matches the typical triboelectric nanogenerator behavior. The maximum power density of 35 μW was reached at a load resistance of 20 MΩ, which created an optimal balance between the voltage and current output as shown in Figure 6a. Quantitative values of key electrical performance parameters of the developed CLP-TENG are presented in Table S1. The CLP matrix showed effective charge transport pathways because it produced high power output levels, with which EDS detects mineral ions (K^+^, Cl^−^, P^5+^, and Mg^2+^) that enhance the interfacial conductivity and charge stability during multiple operation cycles. The change in power density of CLP-TENG with applied load resistance was also measured and it reached a maximum value of 0.0221 W·m^−2^ at 80 MΩ as shown in Figure S9.

The I–V characteristics showed non-linear behavior, which proved that triboelectric processes operate beyond ohmic limits, as shown in Figure 6b. The CLP-TENG generated higher current values at lower external resistance values but produced a lower voltage because it operated through capacitive coupling and dynamic charge redistribution that occurs during contact–separation TENG operations. The device operates stably through Maxwell’s displacement current theory because its output depends on time-dependent polarization and surface charge density instead of direct ohmic relationships. The capacitive and dielectric behavior of the CLP-based triboelectric interface becomes evident through this non-linear output pattern.

During the contact–separation cycle, triboelectric charges were generated due to the difference in electron affinity between PTFE (tribo-negative) and CLP (tribo-positive). Upon contact, electrons transferred from CLP to PTFE, creating opposite surface charges; the subsequent separation induced a time-dependent electrostatic potential difference that drove the displacement current through the external circuit, as governed by Maxwell’s displacement current principle rather than Ohmic conduction. This dynamic process, characterized by charge accumulation, electrostatic induction, and alternating potential, produced the observed non-linear I–V behavior typical of contact–separation TENGs, where the current flow depends on the surface charge density and dielectric properties rather than steady-state electron transport. The dominance of oxygen-rich functional groups in CLP further enhances charge trapping and polarity, reinforcing the triboelectric mechanism. The standard conduction process of a TENG device operating in contact separation mode is represented in the schematic diagram of Figure S5. CLP-TENG achieved superior output performance because its structural and chemical properties worked together to produce enhanced results. The natural pores of CLP assisted in expanding contact areas which enabled a better charge accumulation at multiple surface locations. The FTIR analysis shows that hydroxyl and carboxyl groups function as powerful electron donors which help transfer electrons toward the electron-accepting PTFE surface. The flexible nature of CLP enables it to deform when impacted, which creates optimal air-gap dimensions for better charge storage. The electron-donating CLP (work function ≈ 4.9 eV) and electron-accepting PTFE (≈5.3 eV) create an interfacial potential difference which enabled electron flow when the materials contact and separate. The open-circuit voltage (Voc) can be calculated using the formula Voc = σd/ε_o_, (where σ represents the surface charge density, d represents the separation distance, and ε_o_ represents the vacuum permittivity). The porous structure of CLP increased the effective distance between surfaces which resulted in a higher voltage output. The research shows that CLP-TENG achieved its high triboelectric performance because of its combination of a porous structure and polar functional groups and suitable energy-level alignment.

**Figure 7:**
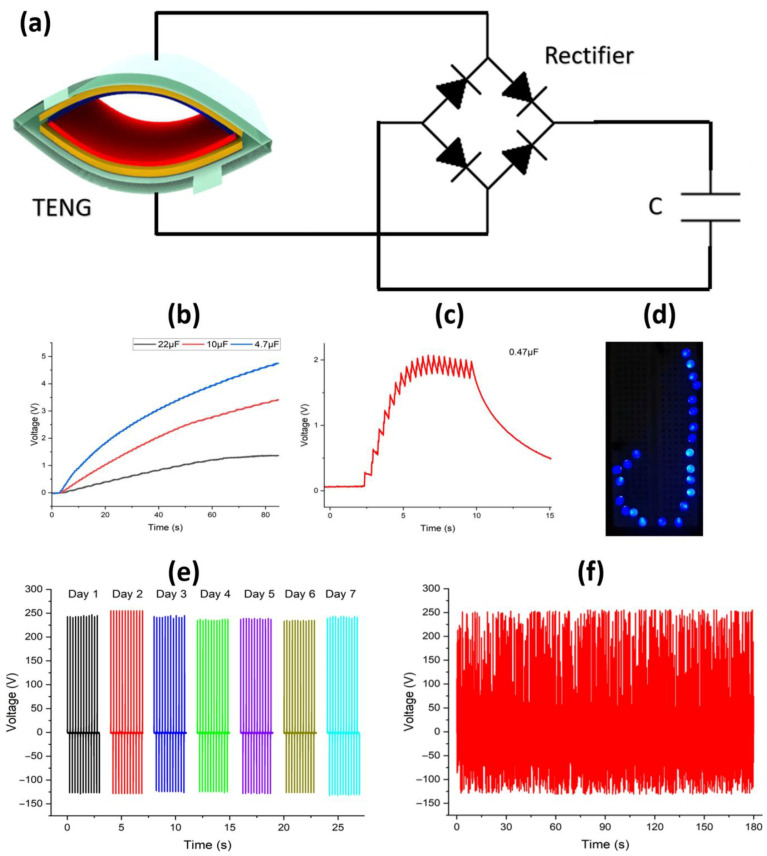
(**a**) Schematic diagram of bridge circuit attached with the TENG to convert AC electrical output to DC for powering microelectronic devices. (**b**) Charging of multiple commercially available capacitors. (**c**) Complete charging and discharging behavior of 0.47 µF capacitor. (**d**) Powering of 23 LEDs through the energy harvesting of CLP-TENG. (**e**) Highly stable results of electrical output of developed CLP-TENG for 1 week continuously. (**f**) Magnified stability behavior of output voltage generated by CLP-TENG during mechanical energy harvesting through linear motor into AC electrical output.

The CLP-TENG produced an alternating output which was converted into direct current (DC) through a bridge circuit, as shown in the schematic diagram of Figure 7a, to charge capacitors with different capacitance values (4.7, 10, and 22 μF) and power 23 light emitting diodes (LEDs). The original AC output was converted into DC (required for powering microelectronics) by using a DF06S full-bridge rectifier circuit (Vishay, Malvern, PA, USA) (Figure S4), with its electrical characteristics obtained from the manufacturer’s datasheet. The charging process showed that smaller capacitors reached their maximum voltage levels before larger capacitors did because the power input remained constant. The 4.7 μF capacitor reached 5 V within 80 s, but the 22 μF capacitor only reached 1.5 V during the same time, as shown in Figure 7b. CLP-TENG demonstrated the ability to provide power to small devices and store energy for extended periods through its continuous operation. The 0.47 μF capacitor showed a steady voltage rise to 2.1 V before discharging while maintaining stable AC–DC conversion patterns through periodic contact–separation events as shown in Figure 7c. Another practical application of powering microelectronics through the developed CLP-TENG was also demonstrated, as shown in Figure 7d. Since the developed CLP-TENG is not a commercial device, testing the reliability and repeatability of its results was also an important aspect of this research work. We operated our system under identical driving conditions to evaluate its long-term operational stability, as shown in Figure 7e,f. The live video demonstration of powering blue color LEDs through CLP-TENG tapping is presented in Video S1. The open-circuit voltage (Voc) showed no signs of degradation because it maintained its original amplitude and waveform structure. The surface charge density (σ) and effective contact area stayed constant during prolonged operation, which preserved the direct relationship between V_OC_ and σ. The CLP-TENG system demonstrated outstanding durability and mechanical resistance because daily tests performed for seven days resulted in no significant performance degradation or noise increase.

### 3.3. Humidity Sensing Characteristics

The CLP-TENG showed humidity-dependent behavior when it was tested under different relative humidity (%RH) conditions in a controlled environment. The Voc showed a steady and reversible decrease when relative humidity (RH) levels were increased. The device showed its ability to detect environmental moisture through this controlled voltage response. The CLP surface became more conducive because water molecules were adsorbed to its hydrophilic functional groups which changed the way charges interacted at the triboelectric interface. The adsorbed water created a thin conductive layer containing mobile ions (H^+^ and OH) that reduced surface charges and created paths for charge dissipation. The surface charge density (σ) decreased, which resulted in a directly proportional decrease in Voc according to the equation Voc ∝ σ. The schematic diagram of the standard charging/discharging process of CLP-TENG operating in contact–separation mode is shown in Figure S5.

**Figure 8:**
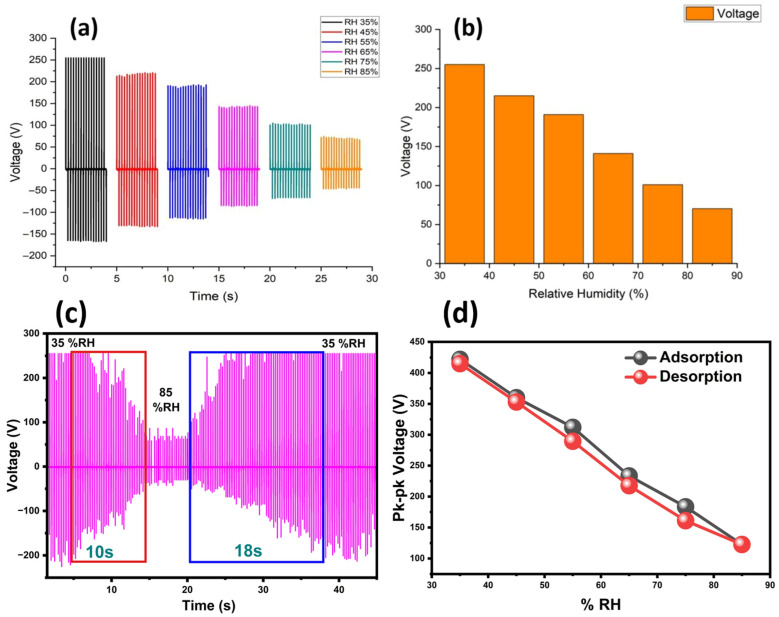
Self-powered humidity response exhibited by CLP-TENG: (**a**) continuous decrease in output voltage with increasing %RH with high stability evident through multiple strikes at one value of %RH; (**b**) specific average values of output voltage produced by CLP-TENG at each specific value of %RH; (**c**) reaction times of CLP-TENG with varying %RH; and (**d**) hysteresis of adsorption/desorption curves for self-powered-CLP-TENG-based humidity sensor.

The FTIR and EDS results showed that the CLP material contained many hydroxyl (–OH) and carboxyl (–COOH) groups and mineral ions (K^+^, Cl^−^, and P^5+^), which made it highly responsive to humidity. The CLP attracted water molecules through strong hydrogen bonding between its polar groups which increased the local dielectric constant and changed the potential difference between the CLP and PTFE layers. The ionic species present in the CLP enhanced the ionic conductivity when they absorbed water, which affected the way in which charges are retained in the system. The CLP-TENG device showed a consistent voltage reduction when %RH levels rose because water adsorption created changes in the dielectric properties and in the overall charge transport mechanism. The CLP surface produced a maximum voltage output of 255 V when operating at humidity levels between 30% and 50% RH. The triboelectric charges become significantly screened by adsorbed moisture when RH reaches 90%, which results in a Voc reduction to half of its original value, as shown in Figure 8a. The CLP-TENG showed excellent performance as a humidity sensor because it can detect environmental changes over a wide range while maintaining high precision in its measurements, as shown in Figure 8b. We also measured the sensitivity of our device by using the mathematical expression of S=(ΔV/ΔRH) (reported in V/RH% or voltage change per RH%) and found its value to be equal to 5.988 V/%RH. The detailed variation in the peak-to-peak output voltage with changing %RH is provided in Table S2. We also calculated and plotted the reaction times of self-powered humidity sensing that resulted in a fast response time of 10 s and reaction time of 18 s (by varying %RH from 35% to 85%) as shown in Figure 8c. The hysteresis curve between the adsorption and desorption of the self-powered CLP-TENG humidity sensor showed little (at higher %RH) or no hysteresis (at low %RH), hence repeating its path both ways as shown in Figure 8d. Because TENG-based humidity sensors are sensitive to temperature variations due to thermally induced voltage, all humidity measurements were performed under constant temperature conditions (25 °C) to eliminate thermal effects. We also developed the 24 h drift response chart for the self-powered CLP-TENG humidity sensor response to illustrate its long-term stability under different %RH conditions as shown in Figure S10. Here, the %24 h drift represented the percentage change in sensor output after 24 h of continuous exposure at a given humidity level. Lower drift values indicated superior stability and minimal signal degradation over prolonged exposure. This metric is critical for assessing the sensor durability and reliability in real-world environments with sustained or fluctuating humidity. Moreover, the stability of the output voltage of CLP-TENG with varying %RH (35% to 85%) recorded over a period of one complete day also exhibited highly stable results as shown in Figure S11.

CLP-TENG operated as a self-powered humidity sensor because it generated an electrical output without requiring any external power source. The CLP’s porous structure enabled fast water penetration into and release out of its surface. The device operated as a flexible environmental sensor with the ability to track %RH in real time while converting mechanical energy into electrical power. The CLP-TENG operated as a dual-function device which generated power from dry environments and operated as a self-sustaining humidity sensor in wet conditions. The device operated as a self-sustaining humidity sensor which made it suitable for applications that need power-free humidity detection in wearable devices and agricultural sensing and smart packaging systems. The successful changes in output voltage of CLP-TENG are demonstrated through a compiled video of Video S2 at different values of %RH. Lastly, a detailed comparison of the output performance of our developed CLP-TENG device with similar recently reported devices is presented in Table S3.

## 4. Conclusions

The research team created a sustainable triboelectric nanogenerator (CLP-TENG) using watermelon rind powder (CLP) as a natural tribo-positive material. The CLP material generated a high electrical output because of its porous structure and flexible microstructure, and abundant surface functional groups allowed efficient charge transfer and production. The device operated steadily through different frequency settings and load resistance levels while showing excellent durability and consistent results throughout extended operation. The CLP-TENG device showed excellent performance as a self-powered humidity sensor through its voltage response, which depended on the humidity levels. The material shows exceptional properties because it combines dielectric strength with ionic conductivity and environmental sensitivity, which most biopolymer-based TENGs lack. The research proves watermelon rind functions as an excellent eco-friendly dielectric material which converts waste into valuable material while creating advanced sustainable triboelectric energy systems. The development of CLP-TENGs for wearable devices and environmental monitoring systems and agricultural applications requires additional research into surface treatment methods for surface stabilization and material protection. The development of self-powered technologies on a scale depends on the research into CLP-TENG integration with wearable devices and environmental monitoring systems and agricultural applications.

## Data Availability

The original contributions presented in this study are included in the article/Supplementary Material. Further inquiries can be directed to the corresponding authors.

## Supplementary Materials

The following supporting information can be downloaded at: https://www.mdpi.com/article/10.3390/polym17233130/s1, Figure S1: Optical images of developed CLP-TENG (a) Area of 16 cm^2^ (b) Gap of 10 mm between both tribo layers in the rest state; Figure S2: (a–f) SEM images of CLP at different resolutions of 150 µm, 100 µm, 30 µm, and 20 µm, respectively showing its highly porous and fibrous structure; Figure S3: Optical image of used customized experimental setup with detailed labeling of each component; Figure S4: Optical image of the used DF06S full-bridge rectifier circuit (Wheatstone Bridge) for converting the original AC output voltage to DC for powering microelectronics; Figure S5: Schematic diagram of a typical TENG device operating in contact-separation mode; Figure S6: (a–d) Effect of striking frequency (from 1 Hz to 4 Hz) on the output current values of CLP-TENG; Figure S7: (a–d) Effect of striking frequency (from 1 Hz to 4 Hz) on the output voltage values of CLP-TENG; Figure S8: (a,b) Reproducibility in terms of output performance of three different developed CLP-based TENG devices under identical conditions; Figure S9: Change in power density of CLP-TENG with applied load resistance, reaching a maximum value of 0.0221 W·m^−2^ at 80 MΩ; Figure S10: 24 h drift response of the developed self-powered CLP-TENG based humidity sensor; Figure S11: Stability of output voltage of CLP-TENG with varying %RH recorded over a period of one complete day; Table S1: Key electrical performance parameters of the developed CLP-TENG; Table S2: Peak-to-peak output voltage of CLP-TENG at varying relative humidity (%RH); Table S3: Output performance comparison of our developed self-powered CLP-TENG based humidity sensor with similar devices; Video S1: Powering LEDs; Video S2: Linear Motor operation under different %RH. Refs [29,41,42,43,44,45] cited in Supplementary Materials.
