# Supplementary material for: Sustainable Cellulose- and Pectin-Rich Triboelectric Nanogenerator for Mechanical Energy Harvesting and Self-Powered Humidity Sensing
Source: Polymers (Basel). 2025 Nov 25;17(23):3130. doi: 10.3390/polym17233130 (PMC12694071; doi:10.3390/polym17233130)
Supplement: Supplementary file 1 [file polymers-17-03130-s001.zip › polymers-3993513-supplementary.pdf]

Supplementary Informaton

Sustainable Cellulose and Pectin Rich Triboelectric Nanogenerator for Mechanical Energy Harvesting and Self-Powered Humidity Sensing

Seongwan Kim [1], Farhan Akhtar [1], Shahzad Iqbal [1], Muhammad Muqeet Rehman [2,*], and Woo Young Kim [1,*]

[1] Faculty of Applied Energy System, Department of Electronic Engineering, Jeju National University, Jeju, 63243 Korea.
[2] Faculty of Electrical Engineering, Ghulam Ishaq Khan Institute of Engineering Sciences and Technology, Topi, KPK, 23640, Pakistan.

***** Correspondence: muqeet.rehman@giki.edu.pk (MMR); semigumi@jejunu.ac.kr (WYK)

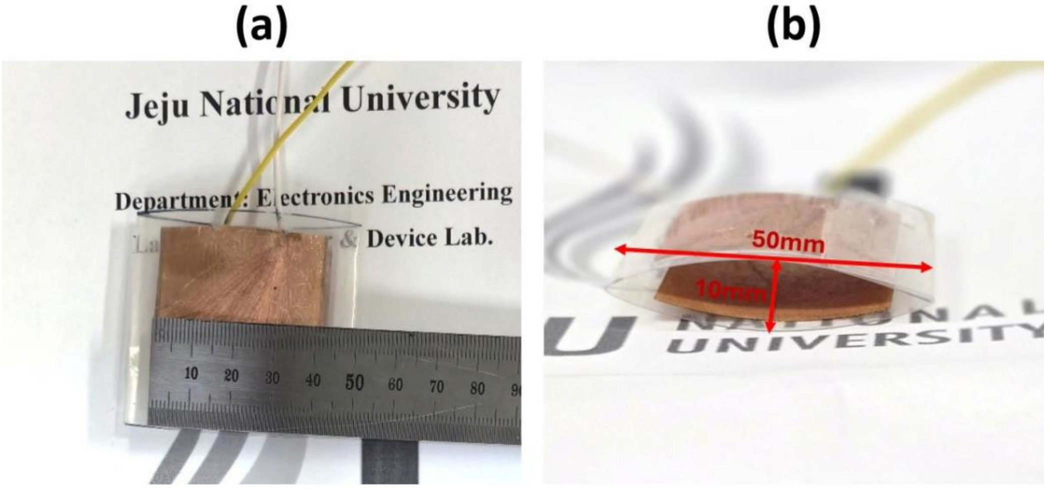

Figure S1. Optical images of developed CLP-TENG (a) Area of 16 cm^2 (b) Gap of 10 mm between both tribo layers in the rest state.

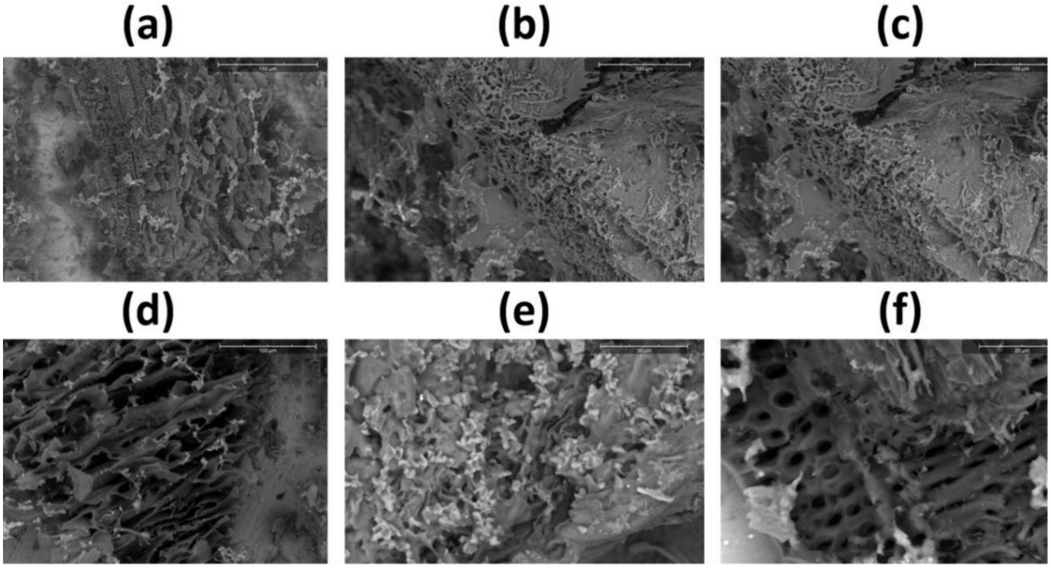

Figure S2. (a–f) SEM images of CLP at different resolutions of 150 μm, 100 μm, 30 μm, and 20 μm, respectively showing its highly porous and fibrous structure.

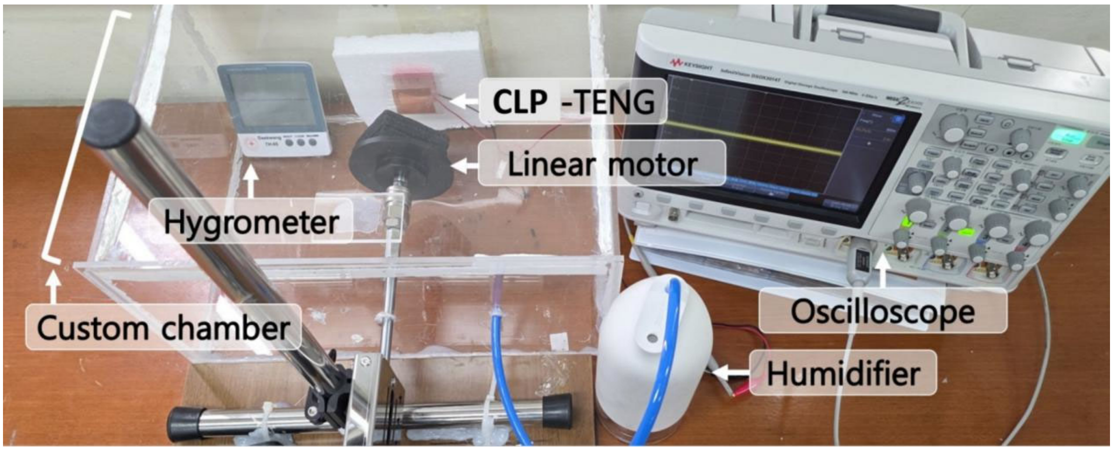

Figure S3. Optical image of used customized experimental setup with detailed labeling of each component.

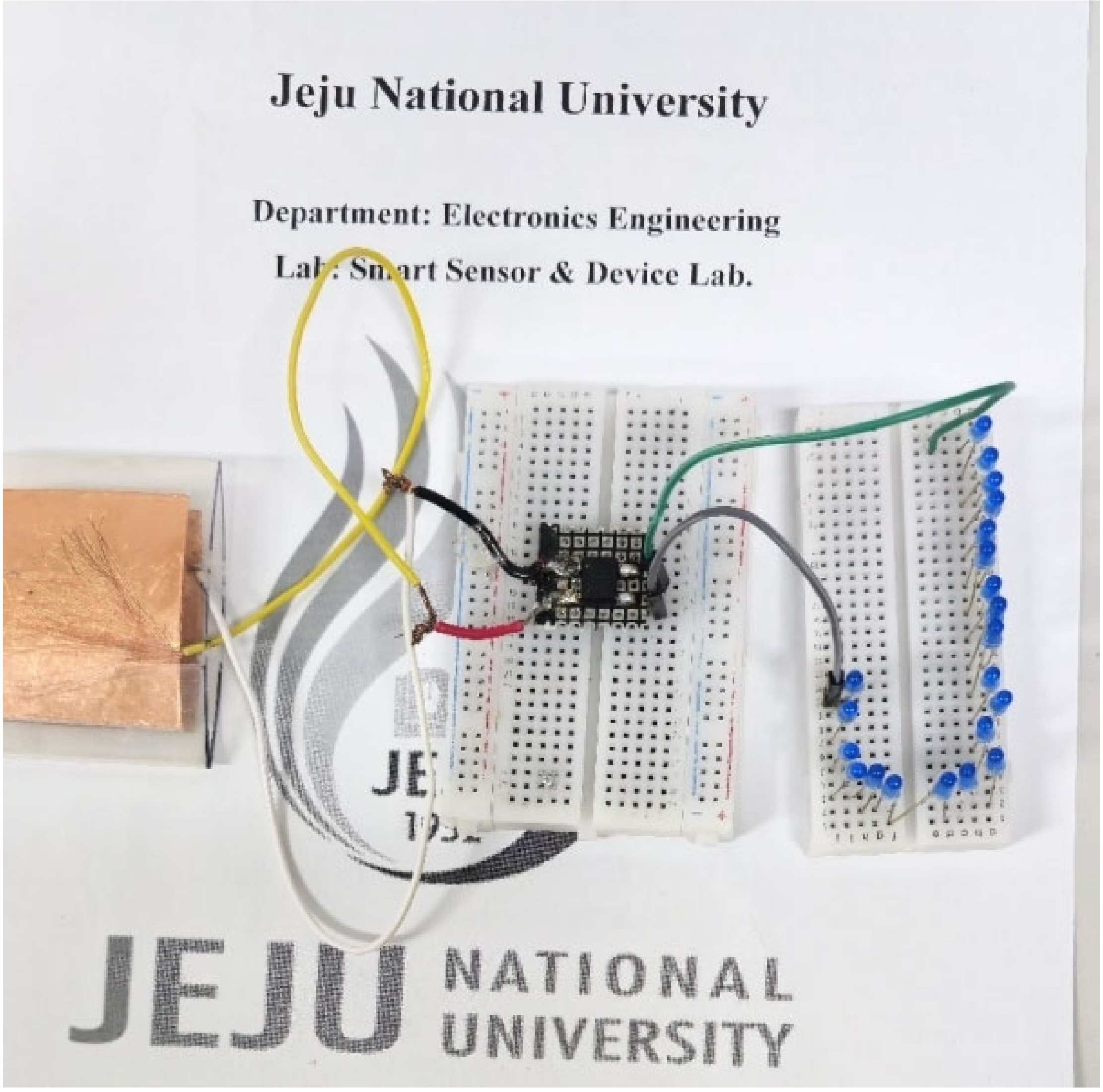

Figure S4: Optical image of the used DF06S full-bridge rectifier circuit (Wheatstone Bridge) for converting the original AC output voltage to DC for powering microelectronics.

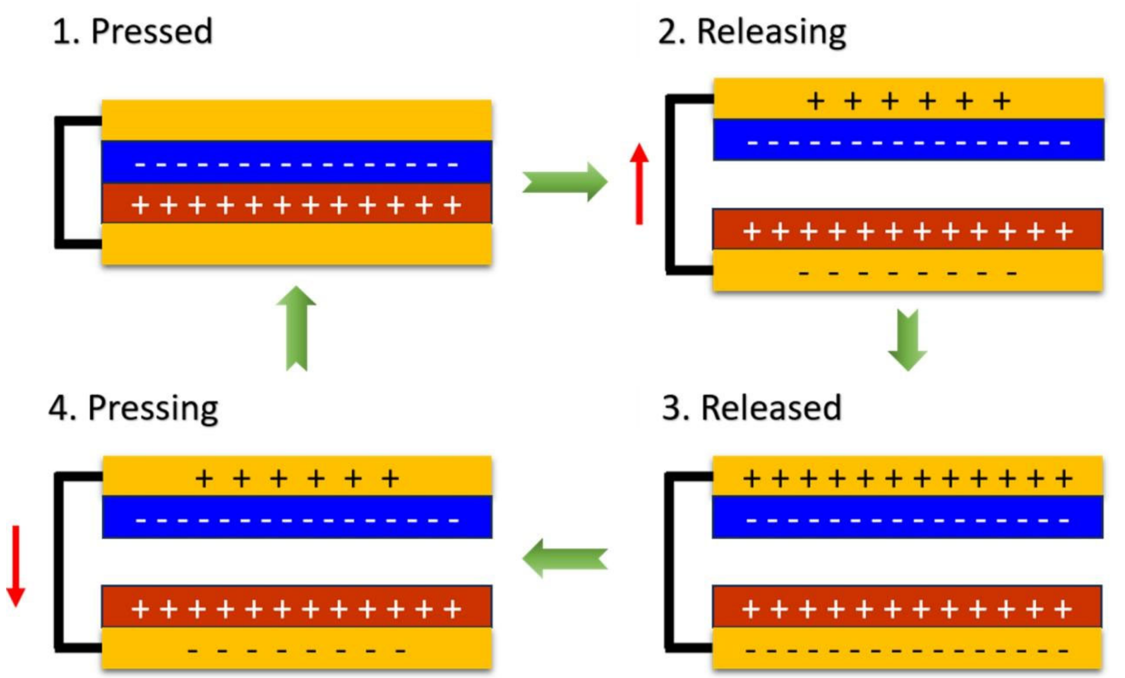

Figure S5. Schematic diagram of a typical TENG device operating in contact-separation mode.

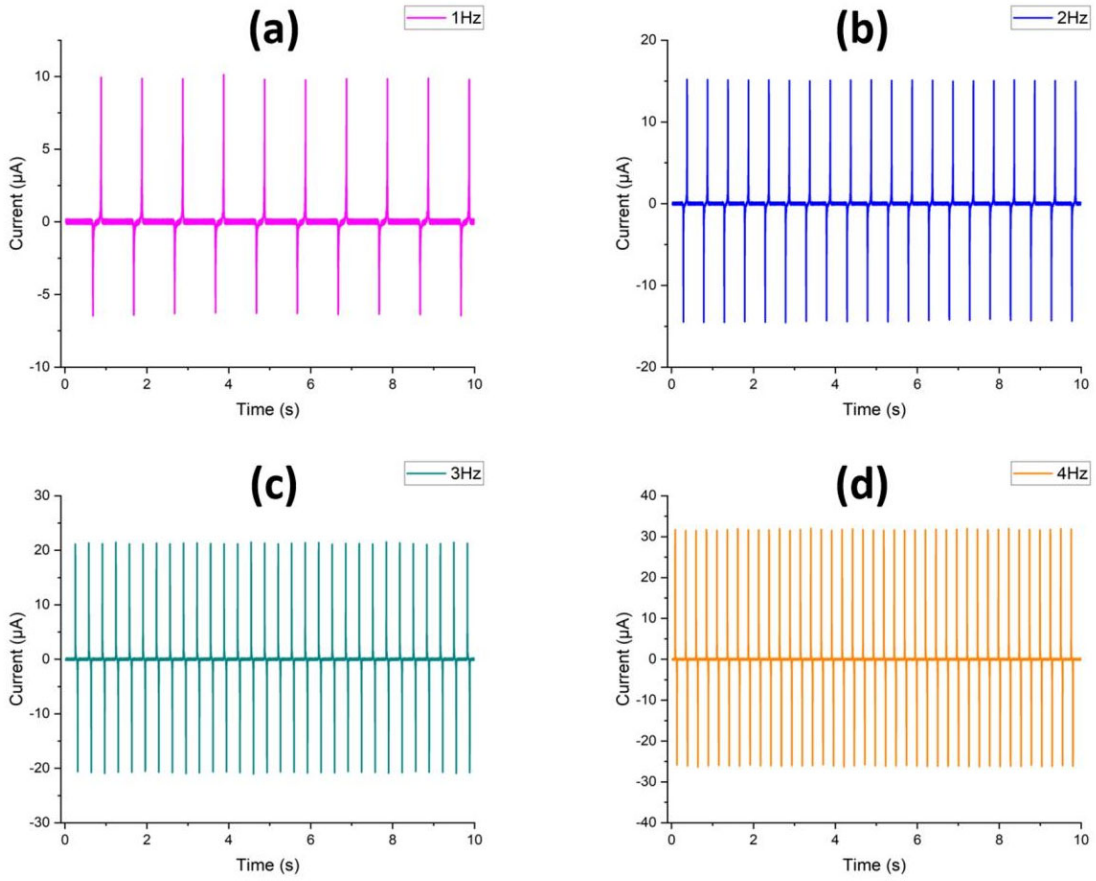

Figure S6. (a–d) Effect of striking frequency (from 1Hz to 4 Hz) on the output current values of CLP-TENG.

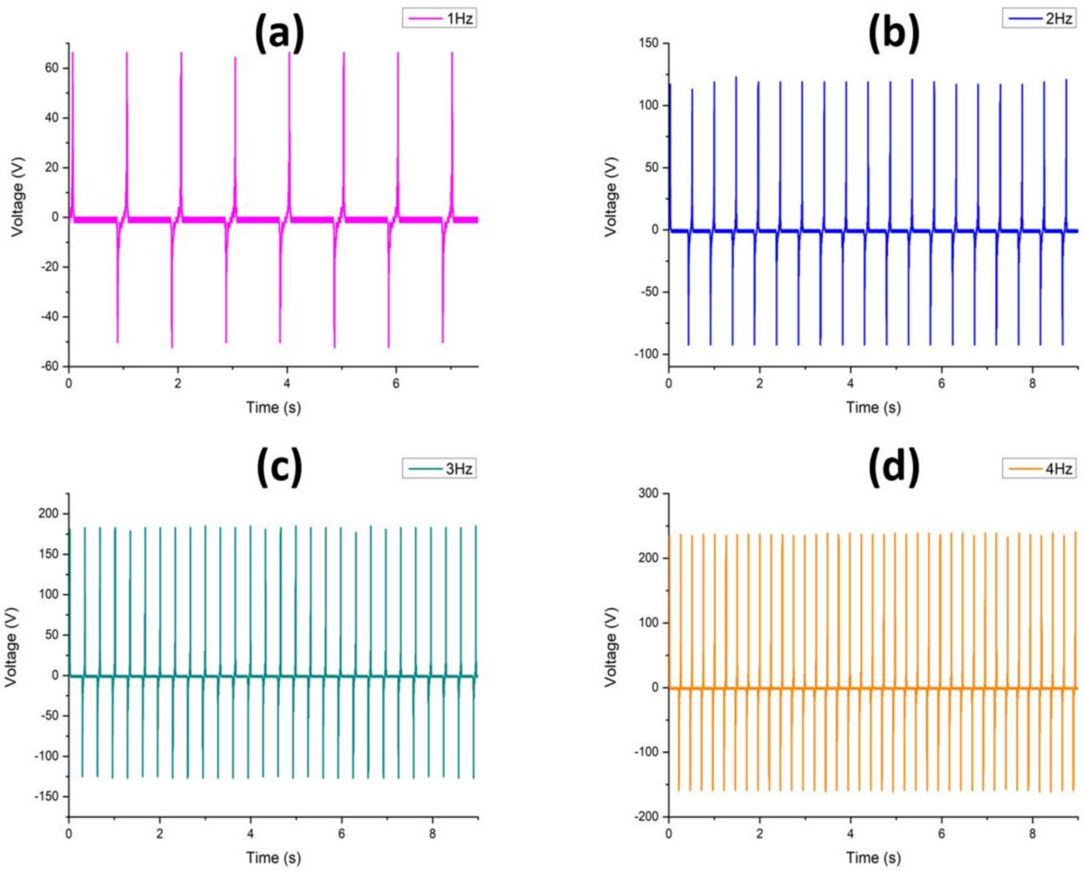

Figure S7. (a–d) Effect of striking frequency (from 1Hz to 4 Hz) on the output voltage values of CLP-TENG.

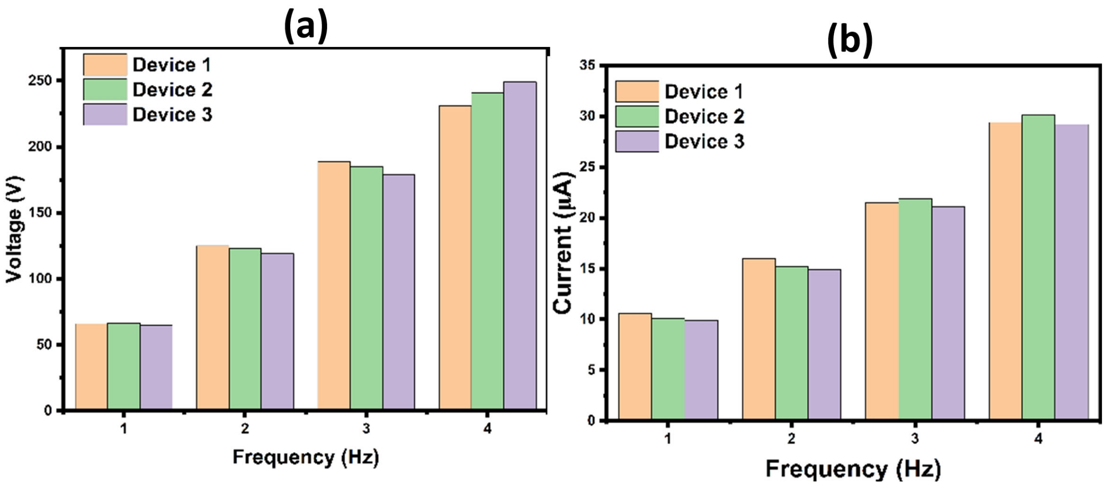

Figure S8. (a,b) Reproducibility in terms of output performance of three different developed CLP-based TENG devices under identical conditions.

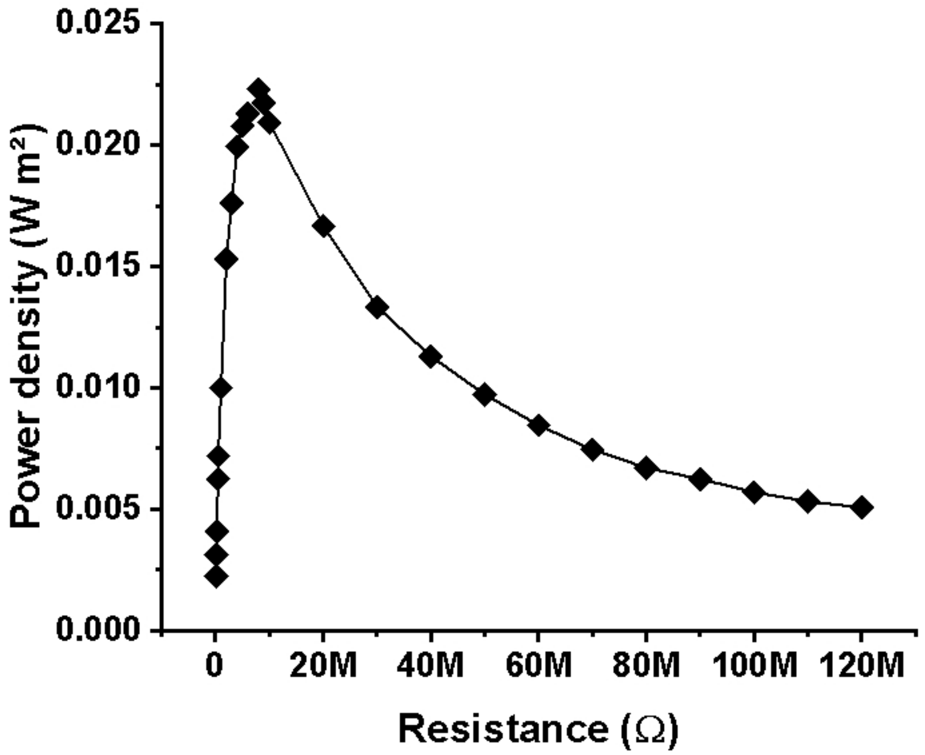

Figure S9. Change in power density of CLP-TENG with applied load resistance, reaching a maximum value of 0.0221 W·m^{-2} at 80 MΩ.

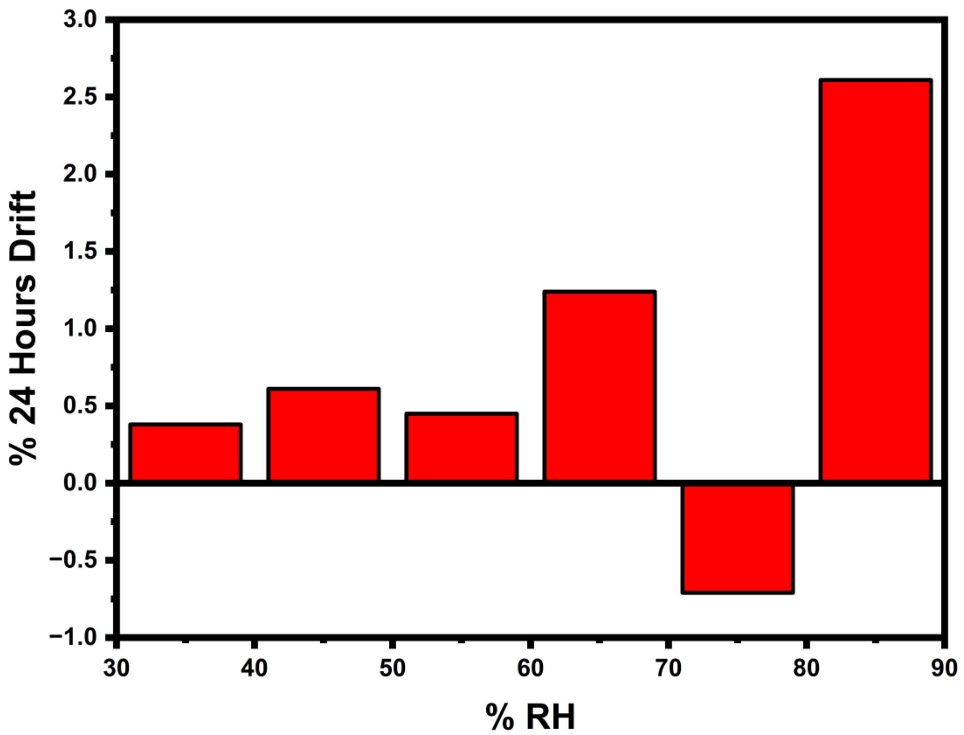

Figure S10. 24 hours drift response of the developed self-powered CLP-TENG based humidity sensor.

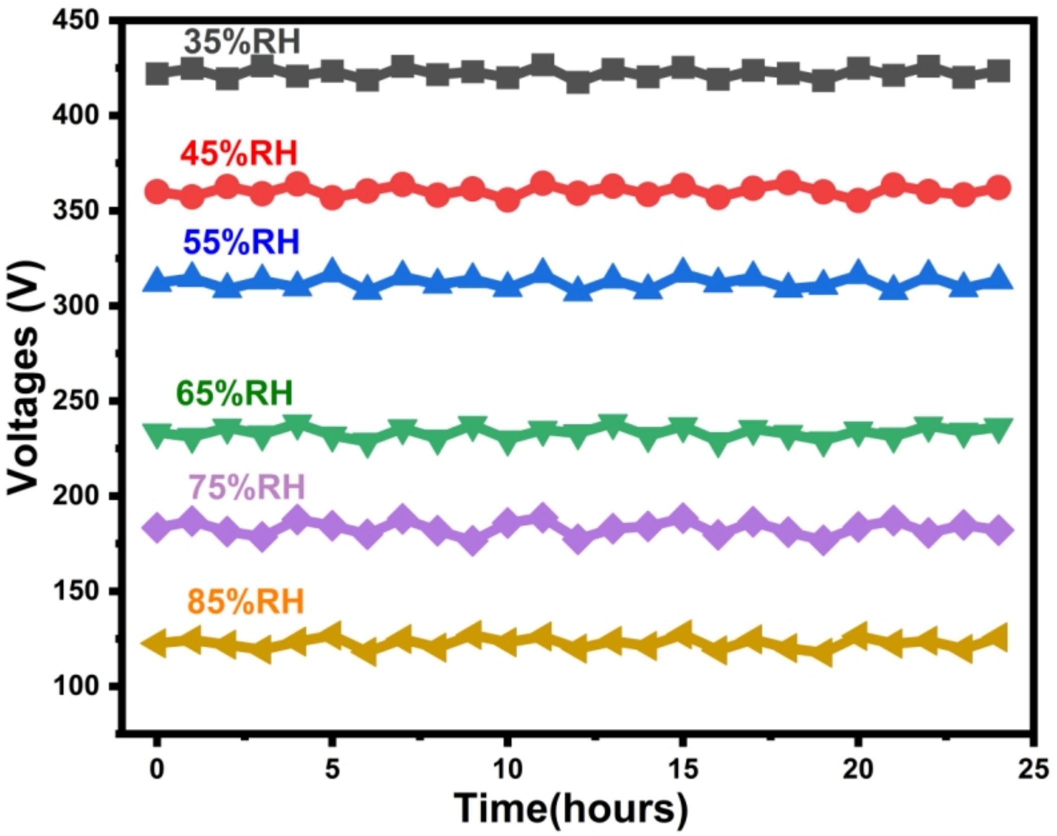

Figure S11. Stability of output voltage of CLP-TENG with varying %RH recorded over a period of one complete day.

Table S1. Key electrical performance parameters of the developed CLP-TENG.

Parameter	Value
V_{OC}	255 V
I_{SC}	35 μA
Q	8.75 μC
σ	0.55 μC·cm^{-2}
J	2.19 μA·cm^{-2}
P_{max}	35 μW @ 20 MΩ

Table S2. Peak-to-peak output voltage of CLP-TENG at varying relative humidity (%RH).

%RH	Pk-pk Voltage (V)
35	422
45	360
55	312
65	233.4
75	183.4
85	122.6

Table S3. Output performance comparison of our developed self-powered CLP-TENG based humidity sensor with similar devices.

Active Material	Sensing Type	Sensitivity	Response time (s)	Recovery time (s)	Operating range (%)
PVA and Parylene C [1]	Resistance change with %RH	0.34 ΔR/%ΔRH	148	110	25 to 98
Hyaluronic acid-induced Nb_2CT_x nanosheets [2]	Voltage change with %RH	NA	15.1	3.4	0 to 72
Chitosan-Based Plasmonic Metal–Hydrogel–Metal Filters [3]	Current change with %RH	0.001–0.0026 A/%RH	1500	NA	10 to 90
Chitosan/Amido-Graphene oxide [4]	Voltage change with %RH	0.16 V/%RH	254	167	18.7 to 91.5
Polypyrrole modified Melamine Aerogel [5]	Voltage change with %RH	1 mV/%RH	32	13	11 to 98
Edible Almond Seed Skin [6]	Voltage change with %RH	0.54 V/%RH	21	14	29 to 100
Citrullus lanatus rind powder [This wrok]	Voltage change with %RH	5.988V/%RH	10	18	35 to 85

Video S1: Powering LEDs (attached with other submission files)

Video S2 Linear Motor operation under different %RH (attached with other submission files)

References:

[1] Jeong, Wooseong, Jinkyu Song, Jihoon Bae, Koteeswara Reddy Nandanapalli, and Sungwon Lee. "Breathable nanomesh humidity sensor for real-time skin humidity monitoring." ACS Applied Materials & Interfaces 11, no. 47 (2019): 44758-44763.
[2] Zhao, Qiuni, Yadong Jiang, Liu Yuan, Zhen Yuan, Boyu Zhang, Bohao Liu, Mingxiang Zhang, Qi Huang, Zaihua Duan, and Huiling Tai. "Hydrophilic hyaluronic acid-induced crumpling of Nb2CTx nanosheets: enabling fast humidity sensing based on primary battery." Sensors and Actuators B: Chemical 392 (2023): 134082.
[3] Jang, Jaehyuck, Kyungnam Kang, Niloufar Raeis-Hosseini, Aizhan Ismukhanova, Heonyeong Jeong, Chunghwan Jung, Byeongsu Kim, Jung-Yong Lee, Inkyu Park, and Junsuk Rho. "Self-powered humidity sensor using chitosan-based plasmonic metal–hydrogel–metal filters." Advanced Optical Materials 8, no. 9 (2020): 1901932.
[4] Liu, Bo-Hao, Guang-Zhong Xie, Cheng-Zhen Li, Si Wang, Zhen Yuan, Zai-Hua Duan, Ya-Dong Jiang, and Hui-Ling Tai. "A chitosan/amido-graphene oxide-based self-powered humidity sensor enabled by triboelectric effect." Rare Metals 40, no. 8 (2021): 1995-2003.
[5] Xiaoqiang, Li, Sun Qian, Kan Yan, Zhu Yanan, Pang Zengyuan, Jin Yang, Li Mengjuan, and Ioannis S. Chronakis. "Self-powered humidity sensor based on polypyrrole modified melamine aerogel." Materials Letters 277 (2020): 128281.
[6] Saqib, Muhammad, Muhammad Muqeet Rehman, Maryam Khan, Shahzad Iqbal, Ahmed Usman Ali, Ghayas Uddin Siddiqui, and Woo Young Kim. "Adaptable self-powered humidity sensor based on a

highly permeable, hierarchically fibrous, and chaotically textured sustainable biowaste." Sustainable Materials and Technologies 44 (2025): e01374.
